# Computed tomography–based analysis of external jugular vein cross–sectional area for vascular access in cats

**DOI:** 10.3389/fvets.2026.1772712

**Published:** 2026-03-04

**Authors:** Daeyun Seo, Sungtak Hong, Min-Su Kim, Taeho Oh

**Affiliations:** 1 Department of Veterinary Clinical Science, College of Veterinary Medicine and Research Institute for Veterinary Science, Seoul National University, Seoul, Republic of Korea; 2 Department of Veterinary Internal Medicine, College of Veterinary Medicine, Kyungpook National University, Daegu, Republic of Korea

**Keywords:** cat, computed tomography, cranial vena cava, cross-sectional area, external jugular vein, morphology

## Abstract

**Introduction:**

This study evaluated the cross-sectional area (CSA) of the external jugular vein (EJV) in cats using computed tomography (CT) and assessed its correlation with body weight. Additionally, the angles between the EJV and cranial vena cava (CrVC) were evaluated.

**Methods:**

This retrospective study analyzed post-contrast CT scans of 27 cats. The CSA of the EJVs was measured at the level of the cricoid cartilage using multiplanar reconstruction, and the angles between each EJV and the CrVC were assessed. Comparisons of EJV CSA and EJV-CrVC angles were performed using paired *t*-tests and equivalence testing with two one-sided test procedures at a 10% equivalence margin. The correlation between EJV CSA and body weight was evaluated using Pearson's correlation coefficient. Intraobserver and interobserver variability were assessed using intraclass correlation coefficients.

**Results:**

Mean CSA of the left and right EJVs was 7.06 ± 3.32 mm^2^ and 6.81 ± 3.26 mm^2^, respectively. Mean angles between EJVs and CrVC were 154.9 ± 8.58 and 152.5 ± 10.71 °, respectively. No statistically significant differences were observed between sides, and clinical equivalence was confirmed within a 10% equivalence margin. EJV CSA demonstrated a significant moderate positive correlation with body weight. Intraobserver and interobserver variability for both CSA and angle measurements were excellent.

**Discussion:**

No significant morphological differences were identified between the left and right EJVs in cats, suggesting that both sides may be considered equivalent for vascular access. Furthermore, because EJV CSA shows only a moderate correlation with body weight, imaging-based evaluation is recommended for optimal catheter size selection.

## 1 Introduction

Intravascular access is essential for the administration of fluids and medications to critically ill patients in both human and veterinary medicine (1–3). In small animals, the external jugular vein (EJV) is preferred for blood sampling and central venous catheterization (2), as well as for interventional procedures such as pacemaker implantation, heartworm extraction, and balloon angioplasty for the treatment of pulmonary hypertension (4–6). This preference is primarily because the EJV is superficially located, easily accessible, and has a relatively large cross-sectional area (CSA) compared with other peripheral vessels (7, 8). In addition, catheter insertion via the EJV into the cranial vena cava (CrVC) follows a natural anatomical course through the right atrium and tricuspid valve, facilitating access to the right ventricular outflow tract (8, 9).

In human medicine, vascular access via the internal jugular vein (IJV) is preferred, with the right side generally favored over the left because the CSA of the right IJV is significantly larger than that of the left, which poses a lower risk of injury to major vessels or the thoracic duct, and provides a relatively straight pathway for easier access to the superior vena cava (10–12). Furthermore, the CSA of the IJV has been shown to correlate significantly with the success rate of central venous catheterization, further supporting the clinical preference for right-sided access (13).

Similarly, in dogs, the CSA of the right EJV has been reported to be significantly larger than that of the left, providing evidence for a preference for right-sided access during central venous catheterization (14). Additionally, the CSA of the EJV was positively correlated with body weight, and lower body weight has been associated with reduced success rates of central venous catheterization (2, 14).

Cats have a relatively smaller body size than dogs and exhibit less interbreed variation in body weight. The right EJV is generally preferred over the left for vascular access in cats (2); however, evidence supporting this practice is limited. Therefore, analyzing the CSA and EJV morphology with respect to vascular access may improve procedural success and reduce the risk of complications. Furthermore, such analysis may help guide the selection of appropriately sized catheters. Studies in human pediatric patients have demonstrated that a high catheter-to-vessel area ratio is significantly associated with an increased risk of thrombosis (15).

Therefore, this study aims to 1) analyze the CSA of the EJV in cats using computed tomography (CT) data and evaluate its correlation with body weight, and 2) assess the angles between the right and left EJVs and the CrVC.

## 2 Materials and methods

### 2.1 Computed tomography data

This retrospective study analyzed CT data from cats referred to a single animal hospital for diagnostic CT examinations between March 2022 and August 2025. All scans were performed using a 64-slice CT scanner (Aquilion 64; Toshiba Medical Systems, Tochigi, Japan). Cats were intubated and maintained under general anesthesia with mechanical ventilation positioned in either ventrodorsal or dorsoventral recumbency. Scan parameters included a slice thickness of 1.0 mm, a tube voltage of 120 kVp, and a tube current of 100 mA. Post-contrast CT scans were obtained after intravenous administration of an iodinated contrast medium (Omnipaque™ 300; GE Healthcare, Oslo, Norway) at a dose of 2 ml/kg.

Eligible cases were identified as those in which 1) CT scans included the head, neck, and thoracic regions; 2) both pre- and post-contrast images were available; and 3) complete signalment, including breed, sex, age, body weight, and final diagnosis, was available. Cases were excluded if 1) CT scans were not acquired in the ventrodorsal position, 2) the margins of the EJVs or CrVC were unclear on post-contrast images, or 3) concurrent conditions potentially affecting venous morphology, such as cervical or thoracic lymphadenopathy or large intra-abdominal masses, were present. Based on these criteria, post-contrast CT data were extracted as digital imaging and communications in medicine (DICOM) files.

### 2.2 Measurement of external jugular vein cross-sectional area and its angle with the cranial vena cava

**Figure 1:**
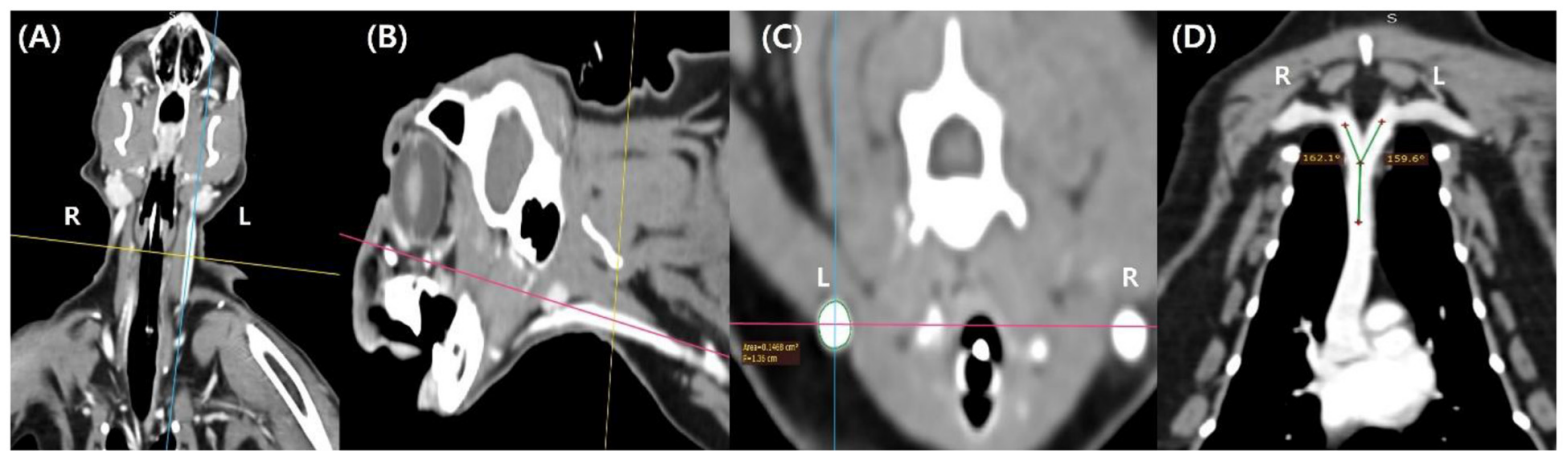
Computed tomography–based measurement of the external jugular vein cross-sectional area and its angle with the cranial vena cava in cats. **(A)** Dorsal plane of a computed tomography scan illustrating the reference planes for measurement. **(B)** Sagittal plane of a computed tomography scan illustrating the reference planes for measurement. **(C)** Transverse plane of a computed tomography scan illustrating the cross-sectional area measurement at the cricoid cartilage level. **(D)** Dorsal plane of a computed tomography scan illustrating the angles formed by the left and right external jugular veins with the cranial vena cava.

The head, neck, and thoracic regions were reconstructed using the multiplanar reconstruction mode of the RadiAnt DICOM Viewer (version 2024.1.0; Medixant, Poznan, Poland). Considering its potential clinical applicability, the CSA of the EJV was evaluated at the level of the cricoid cartilage, which can be identified externally as an anatomical landmark. At this level, the central axis of the EJV was identified in the dorsal and sagittal planes, and a transverse plane perpendicular to this axis was defined for CSA measurement (Figure 1). To clearly delineate the EJV margins, the window level and width were adjusted to 40 and 350–400, respectively, and the CSA was obtained using the closed polygon tool in multiplanar reconstruction mode. After measuring one side, the opposite side was assessed using the same criteria. Subsequently, the angles formed between the central axis of the EJV and that of the CrVC were measured in the dorsal plane using the angle measurement tool in multiplanar reconstruction mode. To assess intraobserver variability, all measurements were performed by a single clinician and repeated twice at a 1-week interval. The mean value of the two measurements was used for analysis. Subsequently, the same parameters were measured by a second clinician, and interobserver variability was assessed based on the measurements obtained by the two clinicians.

### 2.3 Statistical analysis

Statistical analyses were performed using commercially available software (GraphPad Prism 8; GraphPad Software, San Diego, CA, USA, and MedCalc version 23.3.7; MedCalc Software, Ostend, Belgium). Data normality was assessed using the Shapiro—Wilk test, and normally distributed variables are presented as mean ± standard deviation. A preliminary study was conducted by randomly selecting 10 cats, and the bilateral EJV CSAs were found to be similar. As no prior studies were available, the equivalence margin was determined based on the results of the preliminary study and set at 10% of the mean CSA, representing a clinically meaningful difference. The required sample size was calculated using an *a priori* power analysis in G^*^Power (version 3.1; Heinrich-Heine-Universität Düsseldorf, Düsseldorf, Germany). With a power of 0.8 and an alpha level of 0.05, a minimum of 13 pairs was required.

To compare the CSAs of the left and right EJVs, paired *t*-tests were performed. Although the paired *t*-test did not detect a statistically significant difference, this result alone does not indicate that the difference is negligible or clinically meaningful. Therefore, an equivalence test was conducted using the Two One-Sided Tests (TOST) procedure, with the equivalence margin predefined as 10% of the overall mean CSA. Equivalence was concluded when the 90% confidence interval (CI) of the mean difference in CSA was entirely contained within this margin (16). The correlation between the CSA of the EJVs and body weight was assessed using Pearson's correlation coefficient, with correlation coefficients (*r*) categorized as strong (*r* ≥0.7), moderate (0.4 ≤ *r* < 0.7), or weak (0.1 ≤ *r* < 0.4) (17).

The angles formed between the bilateral EJVs and CrVC were compared using the same approach as for the CSA comparison. The intraobserver and interobserver variability of EJV CSA and the angles between the EJVs and CrVC were evaluated using the intraclass correlation coefficient (ICC). ICC values were categorized as excellent (ICC ≥0.9), good (0.75 ≤ ICC < 0.9), moderate (0.5 ≤ ICC < 0.75), or poor (ICC < 0.5) (18). A *p*-value < 0.05 was considered statistically significant.

## 3 Results

CT data from 69 cats were initially reviewed, of which 42 did not meet the inclusion criteria. Consequently, 27 cats were included in the final analysis. The mean body weight was 4.8 ± 1.6 kg, and the mean age was 7.7 ± 4.7 years. The sex distribution comprised 11 spayed females, 10 castrated males, three intact males, and three intact females. Most cats were domestic shorthairs (16/27), followed by Persians, mixed-breed cats, and Siamese cats (2/27 each), with one cat each from the Abyssinian, Munchkin, Scottish Fold, British Shorthair, and Turkish Angora breeds.

### 3.1 Comparison of cross-sectional areas of the left and right external jugular veins

The mean CSA of the left and right EJVs was 7.06 ± 3.32 mm^2^ and 6.81 ± 3.26 mm^2^, respectively (Table 1). Paired *t*-tests indicated no statistically significant difference between the two sides (*p* > 0.05). The equivalence margin was set at 0.69 mm^2^, representing 10% of the mean CSA of the EJVs (6.94 ± 3.26 mm^2^). The 90% CI of the mean difference ranged from −0.516 to 0.005 mm^2^, entirely within the equivalence margin, indicating that the difference between the left and right EJVs is clinically negligible.

**Table 1:** Measurements of the cross-sectional area of the external jugular veins and the angle between the cranial vena cava and the external jugular veins in cats.

**Variables**	**Left EJV**	**Right EJV**	**Bilateral EJVs**
CSA (mm^2^)	7.06 ± 3.32	6.81 ± 3.26	6.94 ± 3.26
Angle (°) between CrVC and EJV	154.9 ± 8.58	152.5 ± 10.71	154 ± 9.66

CrVC, cranial vena cava; CSA; cross-sectional area; EJV, external jugular vein.

### 3.2 Correlation between body weight and cross-sectional areas of the external jugular veins

**Figure 2:**
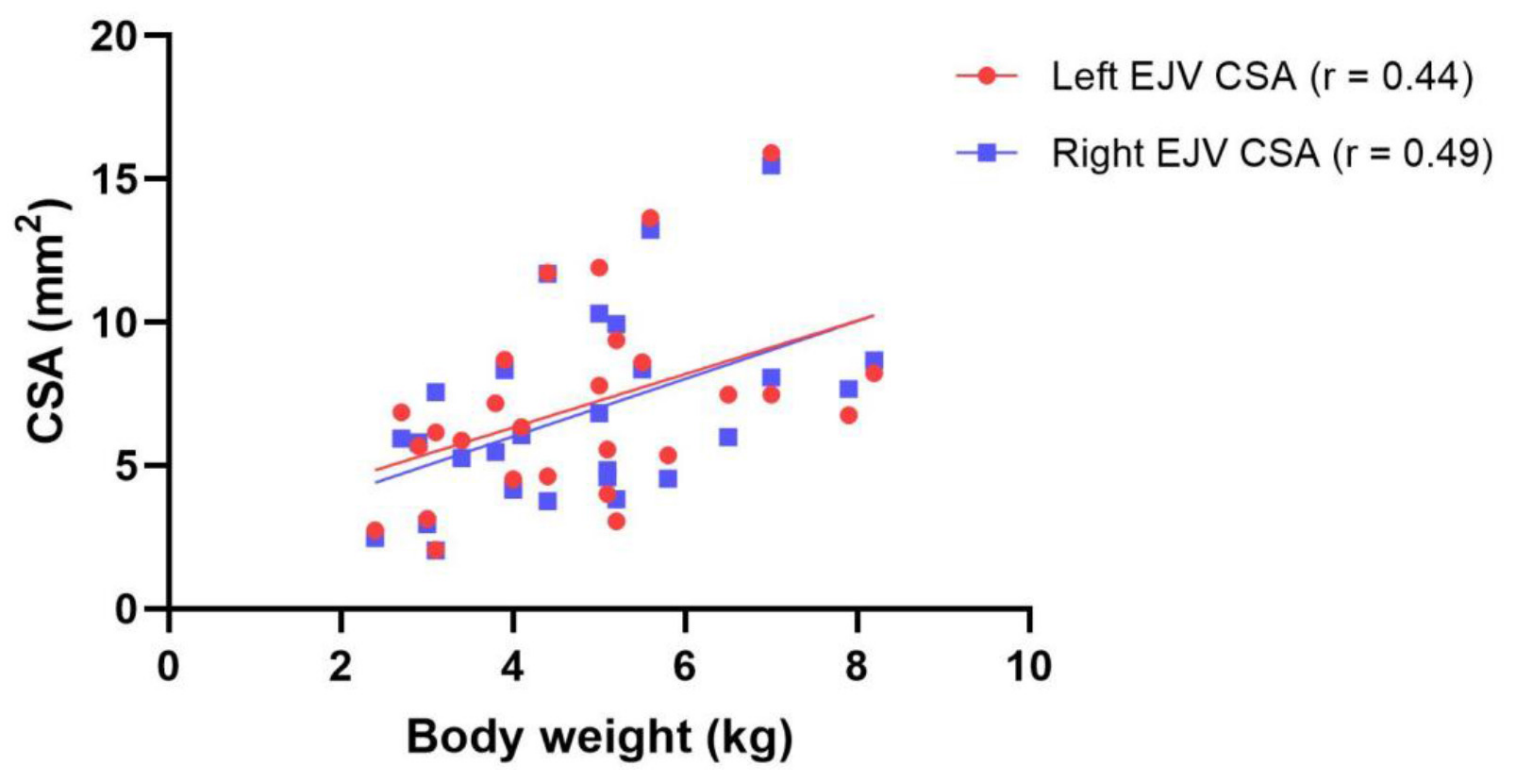
Correlation between body weight and external jugular vein cross-sectional area in cats. Scatter plot showing the relationship between body weight and the cross-sectional area of the left and right external jugular veins in cats. Red circles and the fitted line represent the left external jugular vein, while blue squares and the fitted line represent the right external jugular vein.

Body weight was significantly correlated with the CSA of both the left (*r* = 0.44, *p* = 0.02) and right EJVs (*r* = 0.49, *p* = 0.01), indicating a moderate positive correlation (Figure 2).

### 3.3 Comparison of angles between external jugular veins and cranial vena cava

The mean angle between the left EJV and CrVC was 154.9 ± 8.58 °, while the mean angle between the right EJV and CrVC was 152.5 ± 10.71 ° (Table 1). The overall mean angle between the EJVs and CrVC was 154 ± 9.66 °. The equivalence margin was set at 15.4 °, representing 10% of the overall mean angle between the EJVs and CrVC. The 90% CI of the mean difference ranged from −0.474 to 5.296 °, entirely within the equivalence margin, indicating that the angles between the left and right EJVs and the CrVC are clinically equivalent.

### 3.4 Intraobserver and interobserver variability: cross-sectional area of the external jugular veins and angles between the external jugular veins and cranial vena cava

The ICCs for intraobserver variability were 0.994 (95% CI, 0.986–0.997) and 0.994 (95% CI, 0.988–0.998) for the CSA of the left and right EJVs, respectively. For the angles, the corresponding ICCs were 0.991 (95% CI, 0.981–0.996) for the left EJV and 0.988 (95% CI, 0.973–0.994) for the right EJV.

The ICCs for interobserver variability were 0.992 (95% CI, 0.982–0.996) and 0.988 (95% CI, 0.974–0.995) for the CSA of the left and right EJVs, respectively. For the angles, the corresponding ICCs were 0.958 (95% CI, 0.908–0.981) for the left EJV and 0.976 (95% CI, 0.948–0.989) for the right EJV. These results indicate excellent intraobserver and interobserver variability for both CSA and angular measurements.

## 4 Discussion

This study retrospectively analyzed the CSA of the EJVs and the angles formed between the EJVs and CrVC using CT data from cats. The results demonstrated no clinically significant differences in CSA between the left and right EJVs or in the angles formed between each EJV and the CrVC. In addition, the CSA of the EJVs showed a moderate positive correlation with body weight.

In humans and dogs, the right jugular vein has been reported to have a significantly larger CSA than the left, with differences of approximately 39 and 4.7%, respectively (13, 14). In contrast, this study found that the mean CSA of the left EJV was approximately 3.7% larger than that of the right EJV. Since no statistically significant difference was detected using paired *t*-tests, equivalence testing was subsequently performed using the TOST procedure, confirming that the observed difference was clinically negligible within a 10% equivalence margin. A previous anatomical study of cats reported the diameters of the left and right EJVs at the level of the first cervical vertebra as 3.76 ± 0.70 mm and 3.74 ± 0.73 mm, respectively, although no statistical comparison was performed (19). Assuming circular geometry, the calculated CSAs of the left and right EJVs were 11.10 mm^2^ and 10.99 mm^2^, respectively, with the left approximately 1% larger, consistent with the findings of this study.

In this study, the CSA of the EJVs was positively correlated with body weight. Although statistically significant, the correlation coefficients were 0.44 and 0.49 for the left and right EJVs, respectively, indicating a moderate relationship. Therefore, a predictive formula for EJV CSA based solely on body weight derived through linear regression analysis, may not be suitable for clinical application. In contrast, a previous study in dogs reported a significant correlation between EJV CSA and body weight, with higher correlation coefficients of 0.77 and 0.73 for the left and right EJVs, respectively (14), compared with this study. This discrepancy may be attributed to several factors: 1) the relatively smaller variation in body size among cat breeds compared to dogs, 2) the potential influence of body weight not adjusted for body condition score, and 3) the possible effects of conditions such as intra-abdominal tumors on body weight or vascular diameter. Additionally, because cats with various diseases were included in this study, the results may not fully reflect actual clinical settings. These findings suggest that selecting catheter size for EJV access in cats based solely on body weight may not be appropriate.

Studies in humans have reported that using catheters with diameters larger than that of the vessel increases the risk of thrombosis and reduces the success rate of catheter insertion (15). Accordingly, measuring the CSA of the jugular vein using ultrasound or venography before interventional procedures and selecting a catheter of appropriate size based on these measurements is recommended. Such imaging also enables for the pre-procedural identification of congenital anomalies, including agenesis or hypoplasia of the jugular vein, thereby guiding the selection of an optimal catheter insertion site (9, 18, 20). Similar congenital anomalies have been reported in small animals (9, 21, 22); however, none were observed in this study, potentially due to the small sample size.

The difference in the angle between the jugular vein and the cranial (or superior) vena cava has been reported as a factor contributing to the preference for right-sided access in both humans and veterinary medicine, as the right-sided approach provides a relatively straighter path to the heart, facilitating easier catheter advancement (8, 12). However, in this study, the angle between the EJVs and CrVC was clinically negligible within a 10% equivalence margin, with the mean angle being slightly larger on the left side. This findings suggests that the preference for right-sided access in cats may be influenced by factors other than the CSA of the EJV or its angle with the CrVC, including the risk of thoracic duct or major vessel injury, the presence of venous valves, or operator experience.

This study has several limitations. First, it was a retrospective analysis of cats with various underlying conditions. Although cats with diseases potentially affecting EJV CSA were excluded, the presence of undetected anomalies cannot be completely ruled out. Second, the EJVs are highly compliant and may be easily compressed by positioning aids during CT scanning (23). To minimize such effects, measurements were standardized in the ventrodorsal position; thus, different trends may have occurred in the lateral or dorsoventral positions. Head position is also known to influence IJV CSA in humans, and its potential effect in cats cannot be excluded (24). Third, CT scans were performed under general anesthesia with mechanical ventilation. Mechanical ventilation, the respiratory cycle, and anesthetic agents can affect EJV CSA (25, 26), so the results in conscious cats may differ. Nevertheless, these effects are expected to influence both EJVs similarly and are therefore unlikely to affect side-to-side comparisons. Fourth, information on body condition score was not available in this study, and body weights that were unusually high or low relative to body size may have influenced the correlation with CSA. Future studies are recommended to incorporate body condition scores. Finally, the mean CSA difference between the left and right EJVs was 3.7%, which fall within the predefined 10% equivalence margin and is considered clinically negligible. However, the equivalence test in this study did not specifically assess the 3.7% difference, but rather whether the difference fell within the 10% margin. Therefore, assessing equivalence using a smaller threshold would require additional studies with larger sample sizes to ensure sufficient statistical power.

In conclusion, this study analyzed CT data from cats and demonstrated no clinically significant differences in the CSA of the left and right EJVs or in the angles between the EJVs and CrVC. These findings suggest that similar vascular access can be achieved from either side in cats, and that the general preference for right-sided access is likely attributable to operator experience or other factors rather than morphological considerations. Furthermore, the moderate correlation between body weight and EJV CSA indicates that selecting catheter size based solely on body weight may be inappropriate, and that catheter size should ideally be determined based on imaging evaluation.

## Data Availability

The original contributions presented in the study are included in the article/supplementary material, further inquiries can be directed to the corresponding author.
