# Correction: Computed tomography–based analysis of external jugular vein cross–sectional area for vascular access in cats

**DOI:** 10.3389/fvets.2026.1823856

**Published:** 2026-03-23

**Authors:** Daeyun Seo, Sungtak Hong, Min-Su Kim, Taeho Oh

**Affiliations:** 1 Department of Veterinary Clinical Science, College of Veterinary Medicine and Research Institute for Veterinary Science, Seoul National University, Seoul, Republic of Korea; 2 Department of Veterinary Internal Medicine, College of Veterinary Medicine, Kyungpook National University, Daegu, Republic of Korea

**Keywords:** cat, computed tomography, cranial vena cava, cross-sectional area, external jugular vein, morphology

Author Sungtak Hong was erroneously spelled as Seong-tak Hong.

The original version of this article has been updated.

